# Blocking β_1_/β_2_-Adrenergic Signaling Reduces Dietary Fat Absorption by Suppressing Expression of Pancreatic Lipase in High Fat-Fed Mice

**DOI:** 10.3390/ijms19030857

**Published:** 2018-03-14

**Authors:** Kyunghwa Baek, Danbi Park, Hyo Rin Hwang, Seong-Gon Kim, Heesu Lee, Jeong-Hwa Baek

**Affiliations:** 1 Department of Pharmacology, College of Dentistry and Research Institute of Oral Science, Gangneung-Wonju National University, Gangneung 210-702, Gangwondo, Korea; baekkbone@gmail.com (K.B.); dan20145196@gmail.com (D.P.); 2 Department of Molecular Genetics, School of Dentistry and Dental Research Institute, Seoul National University, Seoul 08826, Korea; shinlmw0728@naver.com; 3 Department of Oral and Maxillofacial Surgery, College of Dentistry, Gangneung-Wonju National University, Gangneung 28644, Korea; kimsg@gwnu.ac.kr; 4 Department of Oral Anatomy, College of Dentistry and Research Institute of Oral Science, Gangneung-Wonju National University, Gangneung 210-702, Gangwondo, Korea; nightsu@gwnu.ac.kr

**Keywords:** propranolol, pancreatic lipase, high fat diet, obesity, β adrenergic receptor

## Abstract

We investigated whether β-adrenergic antagonists attenuates dietary fat absorption through the regulation of pancreatic lipase (PNLIP) expression in pancreatic acinar cells in the context of high fat diet feeding. Male six-week-old C57BL/6 mice were assigned into an ad libitum fed control diet (CON) and a high fat diet (HIGH). Within each diet group, subgroups of mice were treated with vehicle (VEH) or propranolol, a β-adrenergic antagonist (BB). Over 12 weeks, body weight gain observed in HIGHVEH was mitigated in HIGHBB (+103% vs. +72%). Increase in fecal fat amount observed in HIGHVEH was further increased in HIGHBB. Increase in PNLIP expressions observed in HIGHVEH pancreatic tissues was abolished in HIGHBB. PNLIP expression in mouse primary pancreatic acinar cells and 266-6 cell lines increased with isoproterenol treatment, which was blocked by propranolol. Isoproterenol increased PNLIP expression in a cAMP/protein kinase A/ cyclic AMP response element binding protein (CREB)-dependent manner. CREB directly bound to the CRE on the mouse PNLIP promoter and transactivated PNLIP expression. These results suggest that sympathetic activation increases dietary fat absorption through the upregulation of PNLIP expression and that a β-adrenergic antagonist attenuates obesity development partly through the downregulation of PNLIP expression and inhibition of dietary fat absorption in the context of high fat diet feeding.

## 1. Introduction

Obesity and related conditions that account for the metabolic disorders have increased rapidly. Excessive calorie consumption is sensed by the brain, which then triggers regulatory systems to reduce dietary intake and stimulate energy expenditure [1]. High fat diet, which is the major cause of obesity, activates the sympathetic nervous system (SNS) [1]. SNS fibers innervate tissues in almost every organ system, and the SNS is considered to be a mediator of body organ’s cross talk concerning energy metabolism in brown adipose tissue. Activation of β1- or β2-adrenergic receptors (AR) increases lipolysis and energy expenditure [2,3]. A number of in vivo studies have demonstrated that, in addition to β1- and β2-AR, β3-AR mRNA levels are downregulated in genetically obese fa/fa rats [4] and ob/ob mice [5]. These results provide clues that β3-AR function could modulate the development of obesity in these animals. A missense mutation, Trp64Arg, of the β3-AR in obese humans suggests a possible role of β3-AR in obesity and obesity-associated insulin resistance [6]. Based on these clues, SNS-targeted anti-obesity agents that increase the resting metabolic rate or thermogenesis in adipose tissues by enhancing β3-AR signaling have been the subjects of ongoing investigations over the past 20 years. However, clinical intervention efforts to date to prevent and/or treat obesity using a selective β3-AR stimulator/agonist have not successfully controlled the increasing epidemic of obesity [7].

We previously demonstrated that propranolol, a nonselective inhibitor of β1/β2-AR, mitigates increases in body weight and fat mass induced by a high fat diet [8], which is inconsistent with a role for these β-AR in lipolysis and energy expenditure. On the basis that propranolol antagonizes β1- and β2-AR, but not β3-AR [9], these data suggest that β1- and/or β2-AR signaling may have some pro-obesity activity in the context of high fat diet-induced SNS activation. Thus, when β1- and β2-AR-mediated pro-obesity activity was blocked by propranolol, SNS activation can still exert inhibitory effects on weight gain via the remaining β3-AR activity.

Body fat storage is promoted effectively by dietary fat. Ingested dietary fat, most of which is in the form of triacylglycerol, must be converted into fatty acids and 2-monoacylglycerol by pancreatic lipase (PNLIP) before it can be absorbed by the epithelial cells of the small intestine [10]. Thus, inhibiting the digestion and absorption of dietary fat by suppressing PNLIP expression/activity is one effective way of preventing and treating obesity. Beginning with the studies on Orlistat, many investigations have explored agents that can efficiently suppress dietary fat absorption by acting as lipase inhibitors, thereby reducing the uptake of dietary calories. The pancreas is extensively innervated by sympathetic nerves. However, the effect of SNS activation on the pancreatic PNLIP expression and dietary fat absorption has not previously been explored.

In the present study, we demonstrate that in vivo or in vitro activation of β-AR increases PNLIP expression in pancreatic acinar cells, that this effect is attenuated by β-AR antagonists, and that CREB mediates the β-AR-induced PNLIP transcription.

## 2. Results

Over the course of the 12-week study, the control mice (CONVEH, CONBB) consumed 2.85 ± 0.24 g/day of the control diet. HIGHVEH and HIGHBB groups consumed 2.79 ± 0.34 g/day of the high fat-calorie diet. These intakes provided 13.57 ± 0.70 and 16.48 ± 0.93 kcal/day to CON and HIGH mice, respectively. The food intake over the 12-week period was not different between VEH and BB mice within the same calorie status groups.

### 2.1. Blocking Beta-Adrenergic Signaling Attenuated the Increases in Body Weight and Fat Mass Observed with the High Fat-Calorie Diet

**Figure 1:**
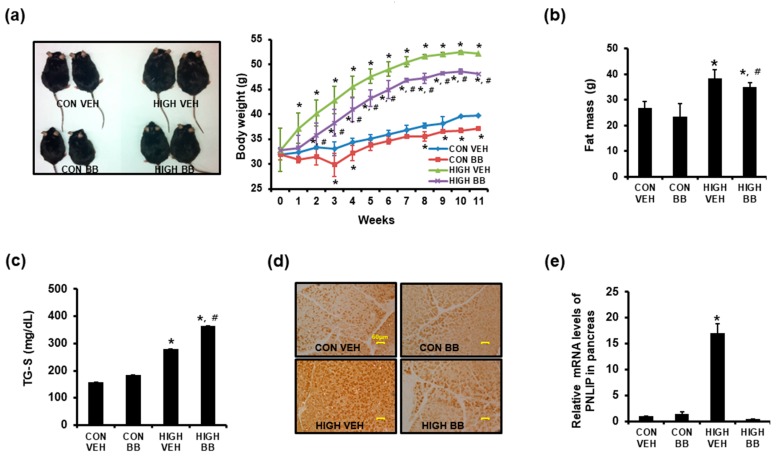
Propranolol attenuated high fat diet-induced body weight/fat mass increase and PNLIP expression. (**a**) The body weights over the 12 weeks of feeding of the high fat diet (HIGH) or the normal diet (CON), in mice administered the vehicle (VEH) or propranolol (β blocker, BB); (**b**) Total body fat mass at week 12. The bars that share the same letters are not significantly different; (**c**) Propranolol further enhanced the high fat diet-induced increase in fecal triglyceride (TG) excretion; (**d**) Pancreatic tissues were immunostained with an antibody to PNLIP (×200). The pancreatic acinar cells positive for PNLIP expression were stained brown; (**e**) The pancreatic levels of PNLIP mRNA were evaluated using quantitative RT-PCR. For all experimental animal groups, *N* = 10. * *p* < 0.05 vs. CONVEH, # *p* < 0.05 vs. VEH within the same diet group. The quantitative RT-PCR data are presented as the means ± S.D. of triplicates.

Over the 12-week experimental period, CONVEH and HIGHVEH mice gained body weight after 12 weeks (+24.06% and +60.70% vs. week 0 within the groups, respectively, *p* < 0.01). Despite the significantly higher body weight gain observed in HIGHBB mice at week 12 (+46.85%) than that of CONVEH, the impact of the high fat-calorie diet on the body weight was significantly attenuated by propranolol (Figure 1a). Even in the control diet-fed mice, propranolol suppressed the age-associated body weight gain. Furthermore, the body fat mass increased over 60% in HIGHVEH mice by week 12, whereas propranolol mitigated this increase (Figure 1b).

### 2.2. Propranolol Further Increased the Fecal Fat Excretion in the High Fat-Fed Mice

PNLIP is a key enzyme that converts the ingested fat into the fatty acids and glycerol. We hypothesized that propranolol downregulates the expression of PNLIP, thereby attenuating dietary fat digestion and absorption in the intestine. We first assessed the quantity of the excreted fecal fat, which reflects how much dietary fat was absorbed. The quantity of fecal fat was higher in HIGHVEH mice than that of CONVEH group (280 vs. 158 mg/dL, respectively) due to the high fat-calorie diet. Interestingly, the fecal fat excretion in HIGHBB mice was much higher than that of HIGHVEH mice (365 vs. 280 mg/dL, respectively) (Figure 1c).

### 2.3. High Fat-Calorie Diet Increased PNLIP Expression in Pancreatic Acinar Cells, and This Effect Was Attenuated by Propranolol

We next examined the levels of the in vivo PNLIP expression using immunohistochemical staining of pancreatic sections (Figure 1d). Compared to those of CONVEH mice, the acinar cells in the pancreatic sections from HIGHVEH mice showed a higher overall PNLIP staining intensity. Propranolol did not induce a significant change in the pancreatic PNLIP expression in control diet-fed mice. However, the increase in the number of PNLIP-positive pancreatic acinar cells observed in HIGHVEH mice was not observed in HIGHBB mice. RT-PCR analyses confirmed increases in pancreatic PNLIP mRNA in high fat diet-fed mice, which increase was blocked by propranolol (Figure 1e).

### 2.4. Isoproterenol Increased PNLIP Expression via Activation of β1- and β2-AR in Pancreatic Acinar Cells

**Figure 2:**
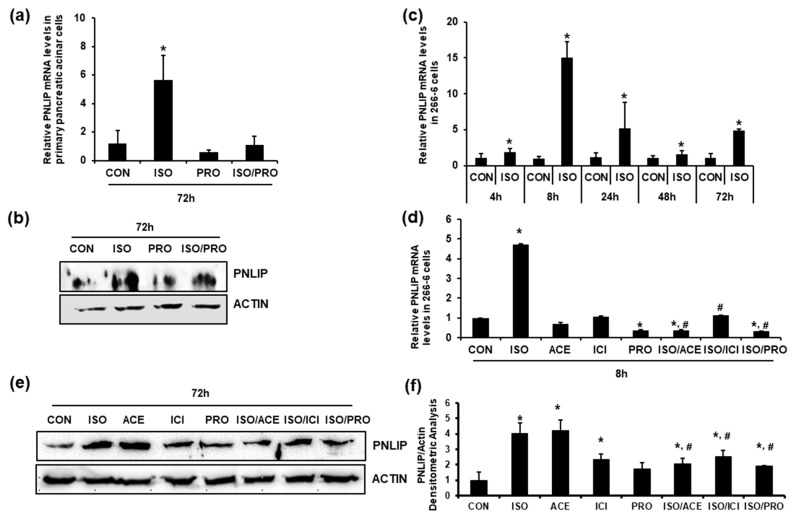
Isoproterenol induced the expression of PNLIP in the pancreatic acinar cells, which was blocked by β1- or β2-adrenergic receptor antagonists. The primary cultured mouse pancreatic acinar cells (**a**,**b**) and the 266-6 pancreatic acinar cell line (**c**–**f**) were incubated with the indicated reagents and PNLIP expression levels were examined using quantitative RT-PCR (**a**,**c**,**d**) and western blotting and densitometric analyses (**b**,**e**,**f**). The quantitative data are presented as the means ± S.D. of triplicates (* *p* < 0.05 vs. CON, # *p* < 0.05 vs. ISO). ISO, 1 μM isoproterenol; PRO, 10 μM propranolol; ACE, 10 nM acebutolol; ICI, 10 nM ICI-118551.

The in vivo results suggested that the high fat-calorie diet increased the dietary fat absorption via SNS activation and the subsequent induction of PNLIP expression in pancreatic acinar cells. Therefore, we confirmed the role β1- and β2-adrenergic signaling with in vitro experiments using primary pancreatic acinar cells (Figure 2a,b) and 266-6 cells (Figure 2c–f). The results demonstrated that isoproterenol increased the levels of PNLIP mRNA and protein. The induction of PNLIP expression by isoproterenol was blocked by treatment with a β1-blocker (acebutolol), a β2-blocker (ICI-118551) or a non-selective β1/β2-blocker (propranolol) (Figure 2d–f.

### 2.5. Isoproterenol Enhanced PNLIP Expression in a cAMP/PKA/CREB-Dependent Manner

**Figure 3:**
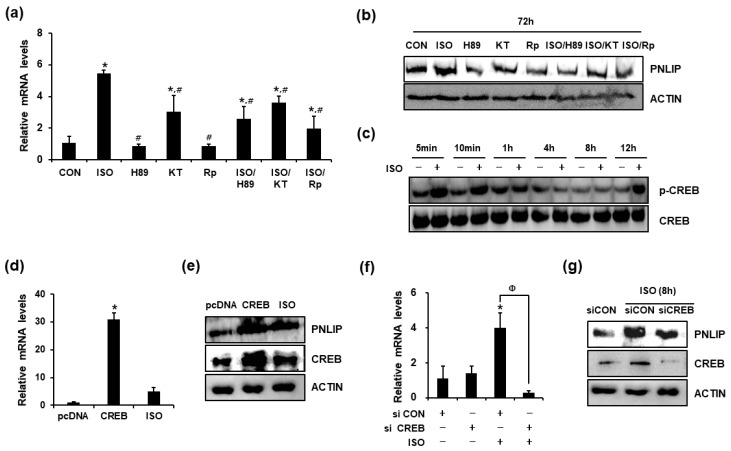
Isoproterenol enhanced the expression of PNLIP in a CREB-dependent manner. (**a**,**b**) The 266-6 cells were incubated for 72 h in the presence of the indicated reagents, and the PNLIP expression levels were examined using quantitative RT-PCR and western blotting analyses. * *p* < 0.05 vs. CON, # *p* < 0.05 vs. ISO. Rp, 10 nM Rp-cAMPs; KT, 5 μM KT5720; H89, 20 μM H89. (**c**) The 266-6 cells were incubated in the presence or absence of isoproterenol for the indicated periods and the levels of CREB and phosphorylated CREB were examined using western blotting analysis. (**d**,**e**) The 266-6 cells were transiently transfected with the pcDNA or CREB expression vector and incubated for 24 h. As a positive control, the 266-6 cells were treated with isoproterenol for 8 h. The levels of PNLIP and CREB mRNA and protein were then examined. * *p* < 0.05 vs. pcDNA. (**f**,**g**) The 266-6 cells were transfected with siRNA targeting CREB (siCREB) or non-targeting control siRNA (siCON) and incubated for 24 h. The cells were then incubated for an additional 8 h in the presence or absence of isoproterenol. The effect of the CREB knockdown on the expression of PNLIP mRNA and protein was then analyzed. * *p* < 0.05 vs. siCON, Φ *p* < 0.05 for the indicated pair.

Activation of β-AR increases cAMP production via activation of adenylate cyclase. Therefore, to characterize the intracellular events involved in the β-adrenergic regulation of PNLIP expression, we examined the effects of cAMP/PKA signaling inhibitors. Rp-cAMPs, KT5720, and H89 significantly downregulated isoproterenol-induced PNLIP expression (Figure 3a,b), indicating that the cAMP/PKA pathway is involved in β-AR-induced PNLIP expression.

Among the downstream elements of cAMP/PKA pathway, CREB is a representative transcription factor. Therefore, we next examined whether CREB mediated the isoproterenol-induced regulation of PNLIP expression in pancreatic acinar cells. When 266-6 cells were treated with isoproterenol, CREB phosphorylation was induced within 5 to 10 min and again at 12 h (Figure 3c). To further investigate the mechanism of CREB-dependent regulation of PNLIP expression, we examined whether CREB overexpression increased PNLIP expression. The efficiency of overexpression was confirmed by quantitative RT-PCR (Supplementary Figure S1a). Overexpression of CREB in 266-6 cells significantly increased the expression levels of PNLIP mRNA and protein (Figure 3d,e). We then knocked down the expression of CREB in 266-6 cells using siRNA. The efficiency of knockdown by CREB siRNA was confirmed by quantitative RT-PCR analysis (Supplementary Figure S1b). The knockdown of CREB decreased the isoproterenol-induced PNLIP expression (Figure 3f,g). These results suggested that the cAMP/PKA/CREB pathway is involved in β-AR-induced PNLIP expression.

### 2.6. CREB Directly Binds to the Mouse PNLIP Promoter and Stimulates PNLIP Transcription

**Figure 4:**
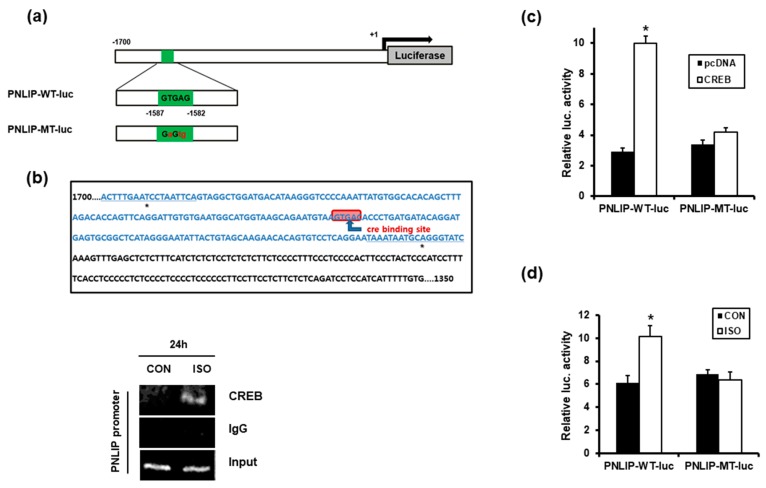
CREB bound to the PNLIP promoter and stimulated PNLIP transcription. (**a**) Schematic illustration of the luciferase (luc.) reporters containing the mouse PNLIP promoter. The highlighted bars show the nucleotide sequences of the putative CRE. (**b**) The 266-6 cells were treated with isoproterenol for 24 h, and the chromatin immunoprecipitation was performed with an antibody to CREB or with normal IgG (lower panel). The amplified DNA region in the PNLIP promoter DNA sequence is highlighted in blue (upper panel). (**c**,**d**) HEK 293 cells were transiently transfected with the indicated plasmids and incubated for 24 h (**c**), or the HEK 293 cells were transfected with the indicated PNLIP promoter reporter and incubated for 24 h in the presence or absence of isoproterenol (**d**). Then, luciferase activity was measured. The data are shown as the firefly luciferase activity relative to the Renilla luciferase activity and represent the means ± S.D (*N* = 6). * *p* < 0.05 vs. pcDNA or CON.

In silico analysis of the mouse PNLIP promoter region demonstrated that one putative CRE is present at −1587 to −1582 bps within the 1.7 kb mouse PNLIP gene promoter region (Figure 4a, Figure 4b, upper panel). Therefore, to examine whether CREB plays a role as a transcriptional activator of the PNLIP gene, we performed a Chromatin Immunoprecipitation (ChIP) assay. The 266-6 cells were treated with isoproterenol for 24 h, and the DNA fragments were immunoprecipitated with an antibody to phospho-CREB or with control IgG. PCR amplification of the PNLIP promoter region encompassing −1587 to −1582 bps revealed that isoproterenol increased the binding of CREB to the PNLIP promoter (Figure 4b, lower panel). We next performed promoter reporter assays to examine whether isoproterenol treatment or CREB overexpression transactivated the PNLIP promoter. Overexpression of CREB significantly increased the reporter activity of PNLIP-wildtype(WT)-luc, whereas the mutations in CRE blocked the CREB-induced luciferase activity (Figure 4c). Treatment of the cells with isoproterenol for 24 h significantly enhanced the PNLIP-WT-luc reporter activity but not the PNLIP-mutant(MT)-luc reporter activity (Figure 4d). Taken together, these results indicated that CREB bound directly to the PNLIP promoter and induced the transcription of the PNLIP gene.

## 3. Discussion

This study is the first to show the relationship between β-adrenergic signaling and PNLIP expression in the context of high fat-calorie diet feeding in mice. Our primary hypotheses were that (1) blockade of β1- and β2-AR signaling by propranolol would attenuate the dietary fat absorption and the high fat-calorie-diet-induced development of obesity and (2) such an effect would be associated with changes in PNLIP expression in pancreatic acinar cells. Our results were consistent with these working hypotheses and suggested an important role of β-AR signaling in the pancreas in response to excessive fat intake.

Because dietary fat is the major source of unwanted calories, PNLIP is considered to be a therapeutic target for the treatment of diet-induced obesity. A line of research has emerged that investigates various methods to regulate the PNLIP expression or activity. Orlistat, an inhibitor of PNLIP isolated from the bacterium *Streptomyces toxytricini*, was the first FDA-approved anti-obesity drug [11]. Importantly, PNLIP and pancreatic lipase-related protein (PLRP), a homologous protein of unknown function [10], are regulated by both amount and type of dietary fat [12,13,14,15]. Ricketts et al. reported that in rats there is a positive correlation between the amount of dietary fat and the expression levels and activity of PNLIP and PLRP, which is consistent with our results [15]. However, the molecular mechanism by which high dietary fat intake regulates PNLIP expression remains unclear.

The mechanistic role of β-adrenergic signaling in preventing the development of obesity has been investigated but has not yet been fully elucidated. In fact, β1- and β2-AR as well as β3-AR have been reported to be expressed in white adipose tissues, although the expression levels of the β1- and β2-AR forms are relatively low, with a ratio of 3:1:150, respectively [5]. Functional quantification of β-AR subtypes indicates that significant uncertainty exists with regard to the relative roles of the β3-AR as well as the β1- and β2-ARs in mediating the β-AR-activated signal transduction processes in white and brown adipose tissues [5,16,17,18]. However, the potential role of β-adrenergic signaling in PNLIP expression has never been demonstrated. To our knowledge, this is the first study to demonstrate that β1- and β2-adrenergic blockade attenuated high fat-calorie-diet-induced body weight gain through the inhibition of PNLIP induction and dietary fat absorption. These data provide evidence for previously overlooked regulatory roles of β1/β2-AR in body energy metabolism. The results support the potential for β1- and β2- as well as global β-blockers as therapeutic drugs for the prevention of obesity.

Our results demonstrate that PNLIP is a novel target gene of CREB, which is activated by β-AR signaling. First, the ChIP assay results showed that isoproterenol–activated CREB bound to the CRE in the PNLIP promoter in vivo. Second, isoproterenol stimulated PNLIP promoter activity in a CRE-dependent manner. Third, overexpression or knockdown of CREB substantially increased or decreased the expression levels of PNLIP, respectively. These results indicated that phospho-CREB directly bound to and transactivated the PNLIP promoter, thus increasing the PNLIP expression. The high fat-fed mice expressed higher levels of the PNLIP mRNA and protein in the pancreatic tissues. Therefore, it is suggested that under conditions that induce SNS activation—including obesity or chronic metabolic disease—β-blockers would contribute to preventing obesity by suppressing PNLIP expression. To better support the direct involvement of CREB in PNLIP expression, further studies using pancreatic acinar cell-specific CREB-null mice will be necessary. Notwithstanding the results demonstrated in the present study, it cannot be overlooked that several reports in humans show that significantly increased weight (fat) gain occurs in subjects treated with beta-blockers [19,20]. Moreover, negative effects of β blocker treatment such as decreased total energy expenditure have been suggested as causative factors of weight gain [21]. However, our reports deals with growing, normal weight mice fed with high fat diet, i.e., subjects that weight gain is in progress. Rodent and human beta-ARs differ with respect to population and expression in each tissue as well as their ability to be stimulated by beta-AR agonists. In addition, we recently reported that beta-adrenergic blockade attenuates high fat-diet-induced increase in body weight/fat mass only if administered when the weight gain is in progress, but not once obesity has already been established [22]. Further research considering difference of population and tissue distribution in beta receptor types between human and mice, obesity onset, and age is justified.

In summary, blockade of β1/β2-AR by propranolol attenuated the body weight/fat mass gain in mice fed the high fat-calorie-diet. Propranolol decreased PNLIP expression in high fat diet-fed mice, which increased fecal excretion of dietary fat. CREB played a role as a transcriptional activator in β-adrenergic signaling-induced PNLIP expression. This unexpected functional relationship between the sympathetic tone and pancreatic acinar cells provides further evidence for the role of the SNS as a control center in the regulation of body energy metabolism.

## 4. Materials and Methods

### 4.1. Animals and Experimental Design

A total of 40 six-week-old C57BL/6 mice (Orient Bio Inc., Seoul, Korea) were assigned by body weight into four groups of ten animals each: ad lib-fed controls treated with the vehicle (CONVEH) or β-blocker (CONBB), and mice fed a high fat-calorie diet treated with the vehicle (HIGHVEH) or β-blocker (HIGHBB). The mice were fed the assigned diet for 12 weeks. CON mice were fed a control diet (Research Diets #AIN93-M; New Brunswick, NJ, USA) which provides 10% of calories from fat. For the high fat-calorie-diet experiments, the mice were fed a high fat diet (60% of calories from fat), a custom-modification of the AIN93-M diet (#D12492, Research Diets; New Brunswick, NJ, USA) for 12 weeks. The β-blocker groups were administered propranolol ((±)-propranolol hydrochloride, Sigma-Aldrich; St. Louis, MO, USA) via the drinking water (0.5 g/L). The dose of propranolol used was equivalent to that which, in previous studies, effectively mitigated high fat diet-induced bone loss and body weight increase [8]. Mice were housed individually under standard housing conditions (room temperature 22 °C, 12 h/12 h light/dark cycle). Immediately before sacrifice, mice were subjected to whole body dual energy X-ray absorptiometry scans (pDEXA; Stratec, Norland Corp.; Fort Atkinson, WI, USA). All procedures in this study were approved by the Seoul National University Institutional Animal Care and Use Committee (SNU-110531-2; 15 June 2012).

### 4.2. Cell Culture

266-6 cells, a murine pancreatic acinar cell line, were maintained in Dulbecco’s Modified Eagles Medium (DMEM; Hyclone; Logan, UT, USA) supplemented with 10% fetal bovine serum (FBS; Hyclone). Culture plates were coated with 0.1% pig skin gelatin (Sigma, St. Louis, MO, USA) in phosphate-buffered saline (PBS) for 30 to 60 min prior to use. AR42J cells, a rat pancreatic acinar cell line, were maintained in F-12K Medium (Kaighn’s Modification of Ham’s F-12 Medium containing 2 mM L-glutamine and 1500 mg/L sodium bicarbonate; ATCC, Manassas, VA, USA) supplemented with 20% FBS. HEK293T cells were maintained in DMEM supplemented with 10% FBS. Primary pancreatic acinar cells were prepared from C57BL/6 mice as described previously [23]. To examine the role of β-AR and cAMP-dependent protein kinase (PKA) pathway, cells were treated with the following reagents: 1 μM isoproterenol (an agonist of β1-, β2- and β3-AR; Sigma), 10 μM propranolol, 10 nM acebutolol (β1-selective antagonist; Sigma), 10 nM ICI-118,551 (β2-selective antagonist; Sigma), 10 nM Rp-cAMPs (an inhibitor of PKA activation; Enzo Life Sciences; Farmingdale, NY, USA), 5 μM KT5720 (a specific inhibitor of PKA; Enzo Life Sciences) and 20 μM H89 (an inhibitor of PKA; Sigma).

### 4.3. Fecal Fat Analysis

Fecal samples were collected at week 9 (48 h collection) to measure the levels of the fecal triglycerides. The amount of fat in the fecal samples was assessed with the TG-S assay kit (AM157; Asan Pharma Co., Seoul, Korea) according to the manufacturer’s instructions.

### 4.4. Reverse Transcription-Polymerase Chain Reaction (RT-PCR)

To evaluate mRNA expression levels, real time RT-PCR was performed as previously described [8]. Mouse genes and the sequences of the PCR primers used for real time-PCR are shown in Supplementary Table S1. For quantification, GAPDH was used as the reference for the normalization of each sample.

### 4.5. Western Blot Analysis

For protein detection, cells were collected and western blot analysis was conducted as previously described [8]. Pancreatic lipase and actin antibodies, and HRP-conjugated secondary antibodies were purchased from Santa Cruz Biotechnology (Santa Cruz, CA, USA). Phospho-CREB and CREB antibodies were obtained from Cell Signaling Technology (Danvers, MA, USA).

### 4.6. Plasmid Construction and Reporter Assays

The mouse PNLIP promoter sequence (−1700 to +1 bp) was selected for cloning by searching mouse genomic sequences (Available online: http://genome.ucsc.edu/). The PNLIP promoter region was amplified by PCR using mouse genomic DNA as a template and subcloned into the (NheI) sites of pGL3b vector (PNLIP-WT-luc). Primers were obtained from Cosmo Genetech (Seoul, Korea), and the primer sequences were as follows: PNLIP- NheI-F 5′- CTT ACG CGT GCT AGC ACT TTG AAT CCT AAT T -3′; PNLIP- NheI-R 5′- CTC GAG CCC GGG CTA GCA ACG AGC AAA CAC TGA -3′. To produce function-defective reporter constructs (PNLIP-MT-luc) that contain mutations in the putative CRE binding site, site-directed mutagenesis PCR (GTGAC→ GaGtg) was performed from −1587 to −1582 bp (MT). The PCR primers used for the site-directed mutagenesis were as follows: PNLIP- NheI-F 5′- CTT ACG CGT GCT AGC ACT TTG AAT CCT AAT T -3′; PNLIP- NheI-R 5′- CTC GAG CCC GGG CTA GCA ACG AGC AAA CAC TGA -3′; PNLIP- MT-R 5′- TAT CAT CAG GGT CAC TCT TAC ATT CTG CTT ACC ATG C-3′; PNLIP- MT-F 5′- AGC AGA ATG TAA GAG TGA CCC TGA TGA TAC AGG ATG-3′. The PCR products were digested with NheI, and the resulting fragments were ligated into pGL3b to produce the mutant reporters. All constructs were verified by DNA sequencing.

HEK293 cells were transfected with the indicated plasmids using LipofectAMINE™ reagent (Invitrogen, Carlsbad, CA). In each transfection, 0.2 μg of expression vector (CREB or pcDNA3.1), 0.2 μg of promoter reporter and 0.1 μg of the Renilla luciferase plasmid were used as indicated. After 24 h, the cells were incubated in the presence or absence of isoproterenol for 24 h and luciferase activity was measured using the Dual-Glo luciferase assay kit according to the manufacturer’s instructions (Promega; Madison, WI, USA). The relative luciferase activity was calculated after normalizing the transfection efficiency by Renilla luciferase activity.

### 4.7. Immunohistochemistry for PNLIP

Sections of the pancreas (5 μm thick) were immunostained for PNLIP using the avidin-biotin technique: the sections were incubated with 1:100 diluted primary rabbit polyclonal PNLIP antibody (Proteintech; Chicago, IL, USA), then with the biotinylated goat anti-rabbit IgG and finally with the ABC complex (Vectastain Elite ABC reagent; VECTOR; Burlingame, CA, USA).

### 4.8. Chromatin Immunoprecipitation (ChIP)

Cross-linked DNA fragments from 266-6 cells were immunoprecipitated with the phospho-CREB antibody or equivalent concentrations of normal rabbit IgG as a negative control. The DNA was then eluted from the immune complexes and subjected to PCR amplification of the region of the mouse PNLIP gene promoter containing the CRE (−1587 to −1582 bp) using the following primer sets: F 5′-ACTTTGAATCCTAATTCA-3′ and R 5′-GATACCCTGCATTATTTA-3′.

### 4.9. Statistical Analysis

To analyze the body weight values, DEXA variables and fecal fat amounts, two-way ANOVA was performed using the SAS (v.9.3) statistical package. In those cases where a significant omnibus F-test (*p* < 0.05) was obtained, the appropriate post hoc Duncan or LSD test was performed to explore the differences among individual means. The statistical significance of the gene expression levels and reporter activities were assessed using Student’s *t*-test. A *p* value < 0.05 was considered statistically significant.

## Supplementary Materials

Supplementary materials can be found at http://www.mdpi.com/1422-0067/19/3/857/s1.
