# Supplementary material for: Blocking β1/β2-Adrenergic Signaling Reduces Dietary Fat Absorption by Suppressing Expression of Pancreatic Lipase in High Fat-Fed Mice
Source: Int J Mol Sci. 2018 Mar 14;19(3):857. doi: 10.3390/ijms19030857 (PMC5877718; doi:10.3390/ijms19030857)
Supplement: Supplementary file 1 [file ijms-19-00857-s001.zip › pnlip_suppl table.docx]

Supplementary Table 1. Primer Sequences for Quantitative RT-PCR

| *Gene* | *Primer sequence (5’-3’)* |
| --- | --- |
| mGAPDH_Fwd | 5'-TCA ATG ACA ACT TTG TCA AGC-3' |
| mGAPDH_Rev | 5'-CCA GGG TTT CTT ACT CCT TGG-3' |
| mPnlip_Fwd | 5’-CTG GGA GCA GCT GGA AG-3’ |
| mPnlip_Rev | 5’-AGC GGG TGT TGA TCT GTG C-3’ |
| mCREB - Fwd | 5’-AGC TGC CAC TCA GCC GGG TA-3’ |
| mCREB - Rev | 5’-TGG TGC TCG TGG GTG CTG TG-3’ |
| rGAPDH_Fwd | 5’-GTA TTG GGC GCC TGG TCA CC-3’ |
| rGAPDH_Rev | 5’-CGC TCC TGG AAG ATG GTG ATG G-3’ |
| rPnlip_Fwd | 5’-ACT TCG CAC GTT GGC GGT ACC A-3’ |
| rPnlip_Rev | 5’-AAT CTC CAA CAT CCA TAT CAG AGT C-3’ |
| rPlrp_Fwd | 5’-CTA TGA GAA GTT CCA GCA GAA TGA CT-3’ |
| rPlrp_Rev | 5’-CCC CTC AAA TTG ATC AGC ATA GT-3’ |
