# Supplementary figures and images for: Blocking β1/β2-Adrenergic Signaling Reduces Dietary Fat Absorption by Suppressing Expression of Pancreatic Lipase in High Fat-Fed Mice
Source: Int J Mol Sci. 2018 Mar 14;19(3):857. doi: 10.3390/ijms19030857 (PMC5877718; doi:10.3390/ijms19030857)

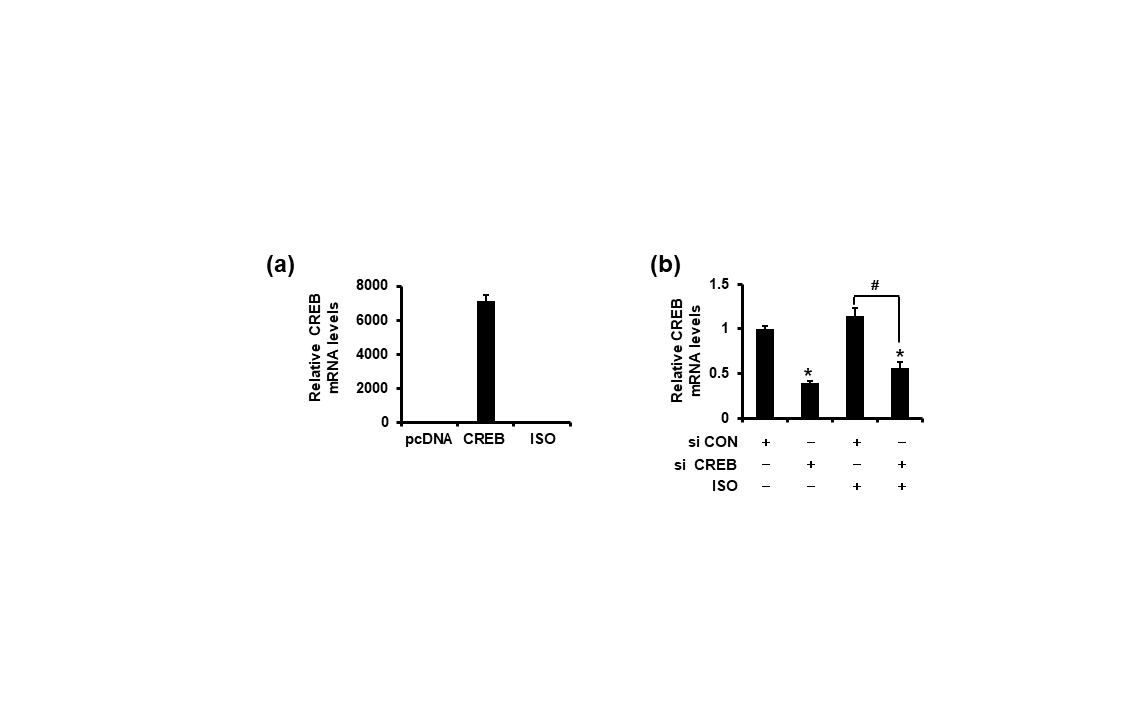

Supplement: Supplementary file 1 [file ijms-19-00857-s001.zip › suppl fig 1.TIF]
